# Efficacy and safety of dronedarone versus placebo in patients with atrial fibrillation stratified according to renal function: Post hoc analyses of the EURIDIS‐ADONIS trials

**DOI:** 10.1002/clc.23765

**Published:** 2022-01-12

**Authors:** Munveer Thind, Wojciech Zareba, Dan Atar, Harry J. G. M. Crijns, Jun Zhu, Hui‐Nam Pak, James Reiffel, Ulf Ludwigs, Mattias Wieloch, John Stewart, Peter Kowey

**Affiliations:** ^1^ Division of Cardiology Lankenau Heart Institute Wynnewood Pennsylvania USA; ^2^ Division of Cardiology University of Rochester Medical Center Rochester New York USA; ^3^ Department of Cardiology Oslo University Hospital Ulleval Oslo Norway; ^4^ Institute of Clinical Medicine University of Oslo Norway; ^5^ Department of Cardiology Maastricht University Medical Centre (MUMC) Maastricht The Netherlands; ^6^ Fuwai Hospital CAMS & PUMC Beijing China; ^7^ Yonsei University College of Medicine Yonsei University Health System Seoul Republic of Korea; ^8^ Division of Cardiology Columbia University Medical Center New York New York USA; ^9^ Sanofi Stockholm Sweden; ^10^ Sanofi Paris France; ^11^ Department of Clinical Sciences Malmö Lund University Malmö Sweden; ^12^ Sanofi Quebec Canada

**Keywords:** antiarrhythmic drugs, atrial fibrillation/atrial flutter, chronic kidney disease, dronedarone, renal function

## Abstract

**Background:**

The use of antiarrhythmic drugs (AADs) in patients with chronic kidney disease (CKD) is complex because impaired renal clearance can cause increased drug levels, and risk of intolerance or adverse events. Due to the propensity for CKD to occur alongside atrial fibrillation/atrial flutter (AF/AFL), it is essential that AAD safety and efficacy are assessed for patients with CKD.

**Hypothesis:**

Dronedarone, an approved AAD, may present a suitable therapeutic option for patients with AF/AFL and concomitant CKD.

**Methods:**

EURIDIS‐ADONIS (EURIDIS, NCT00259428; ADONIS, NCT00259376) were identically designed, multicenter, double‐blind, parallel‐group trials investigating AF/AFL control with dronedarone 400 mg twice daily versus placebo (randomized 2:1). In this post hoc analysis, the primary endpoint was time to first AF/AFL. Patients were stratified according to renal function using the CKD‐Epidemiology Collaboration equation and divided into estimated glomerular filtration rate (eGFR) subgroups of 30–44, 45–59, 60–89, and ≥90 ml/min. Time‐to‐events between treatment groups were compared using log‐rank testing and Cox regression.

**Results:**

At baseline, most (86%) patients demonstrated a mild or mild‐to‐moderate eGFR decrease. Median time to first AF/AFL recurrence was significantly longer with dronedarone versus placebo for all eGFR subgroups except the 30 to 44 ml/min group, where the trend was similar but statistical power may have been limited by the small population. eGFR stratification had no significant effect on serious adverse events, deaths, or treatment discontinuations.

**Conclusions:**

This analysis suggests that dronedarone could be an effective therapeutic option for AF with an acceptable safety profile in patients with impaired renal function.

## 1 INTRODUCTION

Atrial fibrillation (AF) and atrial flutter (AFL) are common cardiac arrhythmias that are often symptomatic and responsible for around one‐third of all arrhythmia‐related hospitalizations.^1^, ^2^, ^3^ Although anticoagulation medication and heart rate control can significantly improve symptoms and decrease risks, restoration of sinus rhythm can further reduce symptoms and greatly improve exercise capacity and quality of life.^4^ One antiarrhythmic drug (AAD), dronedarone, has been proven to reduce cardiovascular hospitalizations or death in people with AF/AFL, complementing the findings of the EAST‐AFNET4 trial of early rhythm control, and highlighting an important therapeutic goal for individuals with AF and AFL.^5^

While amiodarone is a potent AAD, it can cause serious thyroid and systemic toxic side effects in some patients.^6^ A post hoc analysis of the BALKAN‐AF survey found that patients with CKD received amiodarone almost exclusively for rhythm control,^7^ indicating that there is a need for increased understanding of the safety and efficacy of other available therapeutic options. Dronedarone has a similar pharmacological profile to amiodarone, but demonstrates a reduced risk of toxicities, likely due to its increased water solubility, decreased half‐life, and absence of iodine.^3^, ^6^, ^8^, ^9^ Compared to the Vaughan Williams class Ic drugs and sotalol, renal elimination of dronedarone is minimal, with just 6% excreted in urine.^10^ Various randomized controlled trials and real‐world studies have investigated the impact of dronedarone on AF burden and cardiovascular hospitalization.^11^, ^12^ These studies include the European Trial in Atrial Fibrillation or Flutter Patients Receiving Dronedarone for the Maintenance of Sinus Rhythm (EURIDIS, NCT00259428) and the American–Australian–African Trial with Dronedarone in Atrial Fibrillation or Flutter Patients for the Maintenance of Sinus Rhythm (ADONIS, NCT00259376), which were identical trials assessing the safety and efficacy of dronedarone for maintaining sinus rhythm in patients with non‐permanent AF or AFL.^13^ Both trials demonstrated that dronedarone treatment resulted in significantly increased median time to first AF/AFL recurrence, significantly reduced ventricular rate during first AF/AFL recurrence, and significantly reduced rate of hospitalization or death.^13^, ^14^

The European Society of Cardiology has identified a need for research investigating the safety and efficacy of AADs for treating patients also diagnosed with chronic kidney disease (CKD).^15^, ^16^, ^17^ AF rates are particularly high in patients with CKD, ranging from 15% to 40% in patients with end‐stage renal disease, and 16%–21% in patients with stages 3 or 4 CKD.^18^ Additionally, CKD stages 3–5 are present in ~30% of patients with AF,^19^ and more than 50% of patients with AF have an estimated glomerular filtration rate (eGFR) under 60 ml/min.^20^ Comorbid disorders that can facilitate both AF and renal dysfunction, such as hypertension and diabetes mellitus, are commonly represented in AF populations. Typically, patients with CKD are under‐represented or excluded from AAD trials due to an increased risk of proarrhythmic events, particularly in patients with concomitant structural heart disease.^15^, ^18^ As such, there is a significant need for further research regarding the safety and efficacy of AADs for treating people with AF and CKD.

In the EURIDIS‐ADONIS trials, renal function data were collected and eGFR calculated using the Cockcroft‐Gault equation,^13^ however, the CKD‐Epidemiology Collaboration equation (CKD‐EPI) has since become recognized as providing a better estimation of GFR than the Cockcroft‐Gault equation.^21^ Therefore, this post hoc analysis of the EURIDIS‐ADONIS trials calculated eGFR using CKD‐EPI^22^ to investigate the safety and efficacy of dronedarone therapy across a range of eGFR strata.

## 2 METHODS

### 2.1 Study design

The methods for the EURIDIS‐ADONIS trials have been described previously.^13^ EURIDIS‐ADONIS were identical placebo‐controlled, multicenter, double‐blind, parallel‐group trials in which dronedarone efficacy and safety for controlling sinus rhythm in patients with nonpermanent AF/AFL was assessed. Patient eligibility criteria included male or female patients ≥21 years of age, who have had a minimum of one episode of AF/AFL in the preceding 3 months (documented by electrocardiography), and to be in sinus rhythm for at least 1 h before randomization. Exclusion criteria can be found in the supplementary materials. Ethical review boards approved study protocols at each institution and investigations were in accordance with the Declaration of Helsinki.

### 2.2 Baseline evaluation

Baseline evaluations included a medical history, symptom review, cardiovascular examination, assessment of vital signs, 12‐lead electrocardiography (ECG), chest radiography, and laboratory testing. Left atrial size and left ventricular ejection fraction were determined by two‐dimensional echocardiography.

### 2.3 Follow‐up

Following a 7‐day screening period, patients were randomly assigned to either 400 mg of oral dronedarone twice daily or a matching placebo (2:1 ratio). Follow‐up visits to review symptoms, vital signs, and ECGs were performed on days 7, 14, and 21 and at 2, 4, 6, 9, and 12 months. Laboratory tests were repeated on day 21 and at months 4, 9, and 12. Transtelephonic ECGs were performed on Days 2, 3, and 5; at Months 3, 5, 7, and 10; and when symptoms presented. Patients were contacted to confirm the occurrence of one or more symptoms of AF after each ECG. Numbers lost to follow‐up in the EURIDIS trial was 67 (16%) in the dronedarone group versus 25 (12%) in the placebo group. Similar numbers were observed in the ADONIS trial with 81 (19%) patients in the dronedarone group lost to follow‐up versus 36 (17%) in the placebo group.

### 2.4 Study endpoints

The primary endpoint of the study was the time from randomization to the first documented AF/AFL recurrence. An occurrence was classified as an episode lasting for at least 10 min and confirmed by two consecutive 12‐lead ECG or transtelephonic recordings taken 10 min apart. The main secondary endpoints were symptoms related to AF/AFL during 12‐lead ECG recordings or transtelephonic monitoring and the mean ventricular rate during the first recurrence.

### 2.5 Statistical analysis

This was a post hoc analysis on pooled data from the geographically distinct but identically designed EURIDIS and ADONIS trials.^13^ Renal function (eGFR) was calculated using the Cockcroft–Gault equation in the original publication. In this analysis, it was assessed using the CKD‐EPI equation as CKD‐EPI provides a better estimation of GFR than the Cockcroft–Gault equation.^21^, ^22^ Once eGFR was established, patients were grouped by eGFR strata into 30–44, 45–59, 60–89, and ≥90 ml/min subgroups. Time‐to‐events between treatment groups were then compared using log‐rank testing and unadjusted Cox regression. Being an exploratory analysis, *p* values were not adjusted for multiple comparisons. For confirmation purposes, outcomes were also analyzed in eGFR strata classified according to the Modification of Diet in Renal Disease Study Group criteria (Supplementary Table 1A). No difference was observed between the two and so only CKD‐EPI data will be presented herein.

## 3 RESULTS

### 3.1 Baseline characteristics

Patient data from the EURIDIS‐ADONIS trials encompassing 1229 patients were analyzed (Table 1A). Most patients (86%) presented with mild (60–89 ml/min) or mild‐to‐moderate (45–59 ml/min) decreases in eGFR, with females present in higher numbers in groups with worse renal function. As renal function worsened there was a trend towards increasing mean age, and greater prevalence of structural heart disease, coronary heart disease, diabetes, valvular heart disease, presence of pacemakers, and hypertension (Table 1A). Accordingly, CHA_2_DS_2_‐VASc scores increased with decreasing renal function (Table 1A). Left atrial diameter was numerically higher in the subgroup with an eGFR of 30–44 ml/min (43–45 mm) than in those with an eGFR of ≥90 ml/min (40–41 mm). Aligned with the observation of higher numbers of comorbidities, increased use of beta‐blockers, angiotensin‐converting enzyme inhibitors, diuretics, oral anticoagulants, and statins was recorded in groups with greater renal impairment (Table 1B). Digoxin use did not appear to increase with worsening renal function. Amiodarone and sotalol were the most prescribed antiarrhythmic therapies in all eGFR subgroups before randomization, with Vaughan Williams class I AAD use varying between subgroups. No AADs, other than the study drug, were allowed following randomization.

**Table 1A:** Demographic characteristics and cardiovascular disease history

Characteristic and CVD history	eGFR 30–44 ml/min		eGFR 45–59 ml/min		eGFR 60–89 ml/min		eGFR ≥90 ml/min	
	Placebo (*n* = 20)	Dronedarone (*n* = 50)	Placebo (*n* = 99)	Dronedarone (*n* = 234)	Placebo (*n* = 244)	Dronedarone (*n* = 478)	Placebo (*n* = 43)	Dronedarone (*n* = 61)
Age (years), mean ± SD	76.7 ± 6.9	73.3 ± 7.5	67.7 ± 7.9	68.8 ± 8.4	60.4 ± 10.3	61.4 ± 9.5	52.4 ± 11.4	50.5 ± 11.6
Sex, male	9 (45.0)	22 (44.0)	54 (54.5)	136 (58.1)	186 (76.2)	366 (76.6)	30 (69.8)	52 (85.2)
BMI (kg/m^2^), mean ± SD	28.24 ± 6.53	28.83 ± 6.29	29.88 ± 4.92	28.62 ± 4.42	28.88 ± 4.74	28.93 ± 5.38	26.76 ± 5.22	28.74 ± 6.43
CHA_2_DS_2_‐VASC score, mean ± SD	3.5 ± 1.2	3.6 ± 1.5	2.5 ± 1.2	2.7 ± 1.3	1.6 ± 1.3	1.7 ± 1.3	1.1 ± 1.2	0.8 ± 0.8
Baseline heart rate (BPM), mean ± SD	63.3 ± 11.9	63.8 ± 8.7	64.7 ± 11.8	64.3 ± 11.1	62.7 ± 9.9	64.1 ± 10.3	66.7 ± 12.1	66.7 ± 10.2
Structural heart disease	13 (65.0)	31 (63.3)	38 (39.6)	117 (50.6)	93 (38.9)	182 (38.3)	15 (34.9)	15 (25.0)
Coronary heart disease	7 (35.0)	19 (38.0)	23 (23.2)	55 (23.5)	41 (16.8)	111 (23.2)	4 (9.3)	8 (13.1)
Dilated cardiomyopathy	2 (10.0)	5 (10.0)	3 (3.0)	21 (9.0)	23 (9.4)	20 (4.2)	2 (4.7)	3 (4.9)
Hypertension	14 (70.0)	35 (70.0)	68 (68.7)	161 (68.8)	107 (43.9)	275 (57.5)	14 (32.6)	21 (34.4)
Valvular heart disease	8 (40.0)	17 (34.0)	17 (17.2)	55 (23.5)	27 (11.1)	56 (11.7)	9 (20.9)	6 (9.8)
Hypertrophic cardiomyopathy	1 (5.0)	1 (2.0)	5 (5.1)	11 (4.7)	6 (2.5)	11 (2.3)	0	0
Congenital heart disease	0	0	0	7 (3.0)	2 (0.8)	6 (1.3)	1 (2.3)	0
Diabetes	6 (30.0)	9 (18.0)	17 (17.2)	35 (15.0)	20 (8.2)	58 (12.1)	6 (14.0)	2 (3.3)
Left ventricular ejection fraction (%), mean ± SD	54.70 ± 15.65	55.78 ± 14.03	58.37 ± 11.57	56.71 ± 11.85	58.68 ± 10.73	59.77 ± 9.97	59.81 ± 8.21	60.47 ± 8.10
Left atrial anteroposterior diameter (mm), mean ± SD	43.5 ± 6.7	44.9 ± 6.7	41.9 ± 6.1	43.4 ± 7.4	42.6 ± 6.8	42.3 ± 6.7	41.2 ± 8.3	40.6 ± 7.5
Pacemaker	5 (25.0)	6 (12.0)	5 (5.1)	25 (10.7)	7 (2.9)	32 (6.7)	3 (7.0)	0
Implanted cardioverter defibrillator	1 (5.0)	2 (4.0)	1 (1.0)	1 (0.4)	3 (1.2)	3 (0.6)	0	0

*Note*: Data are *n* (%) unless otherwise stated. Placebo group: *n* = 406; Dronedarone group: *n* = 823.

Abbreviations: BMI, body mass index; BPM, beats per minute; CVD, cardiovascular disease; eGFR, estimated glomerular filtration rate; SD, standard deviation.

**Table 1B:** Cardiovascular disease medication history

CVD medication use	eGFR 30–44 ml/min		eGFR 45–59 ml/min		eGFR 60–89 ml/min		eGFR ≥90 ml/min	
	Placebo	Dronedarone	Placebo	Dronedarone	Placebo	Dronedarone	Placebo	Dronedarone
	(*n* = 20)	(*n* = 50)	(*n* = 99)	(*n* = 234)	(*n* = 244)	(*n* = 478)	(*n* = 43)	(*n* = 61)
Betablockers (except sotalol)	13 (65.0)	25 (50.0)	56 (56.6)	138 (59.0)	146 (59.8)	263 (55.0)	22 (51.2)	25 (41.0)
ACE or AII inhibitor	13 (65.0)	30 (60.0)	56 (56.6)	127 (55.0)	104 (43.7)	230 (49.5)	15 (36.6)	19 (35.8)
Digoxin	4 (20.0)	10 (20.0)	24 (24.2)	47 (20.1)	55 (22.5)	81 (16.9)	12 (27.9)	6 (9.8)
Calcium channel blocker (rate lowering)	9 (45.0)	9 (18.0)	20 (20.2)	46 (19.9)	40 (16.8)	74 (15.9)	8 (19.5)	9 (17.0)
Diuretics	11 (55.0)	38 (76.0)	43 (43.4)	93 (40.3)	58 (24.4)	131 (28.2)	11 (26.8)	5 (9.4)
OAC	14 (70.0)	41 (82.0)	74 (74.7)	173 (74.9)	175 (73.5)	322 (69.2)	25 (61.0)	30 (56.6)
Other chronic antiplatelet therapy	12 (60.0)	23 (46.0)	44 (44.4)	90 (39.0)	78 (32.8)	186 (40.0)	18 (43.9)	26 (49.1)
Statins (CYP3A4 metabolized)	6 (30.0)	18 (36.0)	26 (26.3)	60 (26.0)	46 (19.3)	98 (21.1)	6 (14.6)	11 (20.8)
Statins (not CYP3A4 metabolized)	4 (20.0)	7 (14.0)	18 (18.2)	25 (10.8)	31 (13.0)	62 (13.3)	2 (4.9)	7 (13.2)
Moderate inhibitors of CYP3A4	9 (45.0)	10 (20.0)	21 (21.2)	46 (19.9)	42 (17.6)	75 (16.1)	8 (19.5)	9 (17.0)
Previous antiarrhythmic treatment								
Class I	0	2 (4.0)	10 (10.1)	24 (10.3)	20 (8.2)	34 (7.1)	5 (11.6)	5 (8.2)
Amiodarone	10 (50.0)	24 (48.0)	18 (18.2)	52 (22.2)	49 (20.1)	92 (19.2)	8 (18.6)	8 (13.1)
Sotalol	2 (10.0)	7 (14.0)	29 (29.3)	41 (17.5)	56 (23.0)	111 (23.2)	12 (27.9)	20 (32.8)

*Note*: Data are *n* (%). Placebo group: *n* = 406; Dronedarone group: *n* = 823.

Abbreviations: ACE, angiotensin‐converting enzyme; AII, angiotensin II; CVD, cardiovascular disease; eGFR, estimated glomerular filtration rate; OAC, oral anticoagulant.

### 3.2 Time to first AF/AFL (primary outcome)

**Figure 1:**
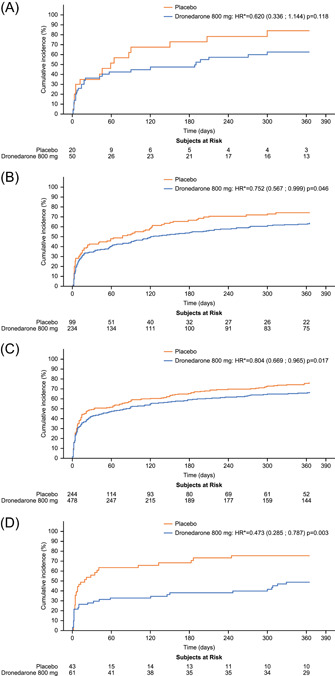
Kaplan–Meier cumulative incidence of adjudicated first recurrence of atrial fibrillation or flutter by eGFR category (A) 30‐44 ml/min, (B) 45‐59 ml/min, (C) 60‐89 ml/min, and (D) ≥ 90 ml/min. *Hazard ratio values determined by Cox regression model

**Figure 2:**
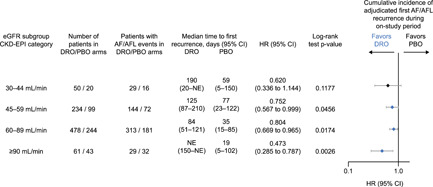
Cumulative incidence of adjudicated first atrial fibrillation/atrial flutter recurrence with dronedarone versus placebo. AF/AFL, atrial fibrillation/atrial flutter; CI, confidence interval; CKD‐EPI, chronic kidney disease‐epidemiology collaboration equation; DRO, dronedarone; eGFR, estimated glomerular filtration rate; HR, hazard ratio; PBO, placebo

Median time to first AF/AFL recurrence was significantly longer in the dronedarone versus placebo group for all eGFR subgroups except the most renally impaired (30–44 ml/min) (Table S1B), whether based on first adjudicated (Figures 1 and 2) or symptomatic episodes (Figure S1). While dronedarone appeared to provide a benefit in time to first adjudicated AF/AFL compared with placebo in all the eGFR subgroups (Figures 1 and 2), this result was only significant in the 45–59, 60–89, and ≥90 ml/min eGFR subgroups, with the most substantial benefit being observed in patients with eGFR ≥90 ml/min. As the hazard ratios for the cumulative incidence of adjudicated first AF/AFL recurrence (Figure 2) were all <1 and not significantly different from one another, no test for interaction was performed. The 30–44 ml/min subgroup demonstrated a trend towards an increased time to first AF/AFL recurrence, but statistical power was limited by the relatively small patient numbers (*n* = 70).

### 3.3 Change in creatinine

A numerical increase in creatinine of between 9.3 and 17.2 μmol/L was observed at Day 7 of treatment in the various eGFR subgroups, whereas creatinine levels had decreased in the 30–44, 45–59, and 60–89 ml/min eGFR subgroups treated with placebo at the same timepoint (Table 2). The numerical increases in creatinine seen at Day 7 in the dronedarone‐treated subgroups compared with the respective placebo subgroups, and the ≥90 ml/min eGFR placebo subgroup were maintained until 12 months at the end of the study.

**Table 2:** Summary of adverse events

	eGFR 30–44 ml/min		eGFR 45–59 ml/min		eGFR 60–89 ml/min		eGFR ≥90 ml/min	
	Placebo (*n* = 20)	Dronedarone (*n* = 50)	Placebo (*n* = 99)	Dronedarone (*n* = 234)	Placebo (*n* = 244)	Dronedarone (*n* = 478)	Placebo (*n* = 43)	Dronedarone (*n* = 61)
Pt with any serious TEAE	7 (35)	17 (34)	27 (27)	53 (23)	53 (22)	85 (18)	13 (30)	8 (13)
Death (any cause)	2 (10)	4 (8)	1 (1)	3 (1)	0 (0)	1 (0)	0 (0)	0 (0)
Pt with any TEAE leading to discontinuation	5 (25)	14 (28)	4 (4)	22 (9)	16 (7)	37 (8)	4 (9)	4 (7)
Creatinine change from baseline (μmol/L), (mean ± SD)								
Baseline	127.8 ± 17.7	130.1 ± 18.4	108.9 ± 13.0	108.5 ± 14.8	93.1 ± 11.9	93.5 ± 12.4	71.9 ± 12.2	75.7 ± 10.1
Day 7	−10.9 ± 16.0	17.2 ± 23.7	−3.0 ± 9.4	9.3 ± 13.0	−0.3 ± 8.7	9.7 ± 11.5	8.0 ± 11.2	13.1 ± 11.9
Day 14	NA	10.8 ± 18.9	−2.8 ± 10.4	9.3 ± 16.8	−1.3 ± 8.4	40.0 ± 115.5	13.4 (NA)	18.5 ± 1.4
Month 2	−8.5 ± 7.8	33.6 ± 33.2	−15.8 ± 6.9	11.2 ± 33.6	−0.2 ± 11.4	8.5 ± 10.4	12.8 ± 6.0	0.8 ± 8.3
Month 6	NA	27.1 ± 30.4	2.7 ± 14.1	7.7 ± 16.1	−0.1 ± 8.7	12.2 ± 20.4	9.2 ± 5.0	13.3 ± 19.2
Month 12	0.6 ± 14.2	13.9 ± 25.7	−2.0 ± 13.9	6.3 ± 20.3	2.3 ± 11.9	9.9 ± 17.0	4.6 ± 11.9	11.3 ± 15.2

*Note*: Data are *n* (%) unless stated otherwise. Patient numbers may vary between time points. Placebo group: *n* = 406; Dronedarone group: *n* = 823.

Abbreviations: eGFR, estimated glomerular filtration rate; NA, not available (none or one patient with data available); Pt, patient; SD, standard deviation; TEAE, treatment‐emergent adverse event.

### 3.4 Heart rate

**Figure 3:**
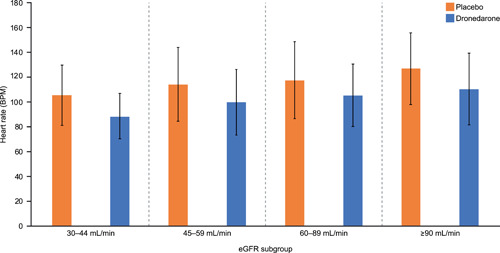
Mean heart rate at first adjudicated atrial fibrillation/flutter. Heart rate values obtained on only one RR interval were not considered. Error bars represent the standard deviation of the mean. BPM, beats per minute; eGFR, estimated glomerular filtration rate

Patients in the 30–44 ml/min eGFR subgroup demonstrated a mean heart rate 3–4 beats‐per‐minute (bpm) slower than the ≥90 ml/min subgroup at baseline (Table 1A), and the use of betablockers was numerically higher in the 30–44 ml/min eGFR subgroup (Table 1B). By the time of first adjudicated AF/AFL, dronedarone treatment was associated with a lower mean heart rate in all eGFR subgroups compared with placebo (Figure 3).

### 3.5 Safety

No notable differences between dronedarone and placebo were observed for incidences of serious adverse events, deaths, and treatment discontinuations among eGFR strata (Table 2).

## 4 DISCUSSION

Achievement and maintenance of sinus rhythm is often a key therapeutic goal for patients with AF/AFL. In this posthoc analysis of the pivotal EURIDIS‐ADONIS trials, dronedarone demonstrated a significantly increased median time to first AF/AFL recurrence versus placebo in all eGFR subgroups except the most renally impaired (30–44 ml/min), where a numerical trend was observed. This was true for both adjudicated and symptomatic recurrences of AF/AFL. The failure to achieve statistical significance in the most renally impaired subgroup despite a trend towards longer time to recurrence may be due to the lower population size relative to the other subgroups as the numerical difference was compelling. Cumulative incidence of AF/AFL recurrence in the placebo group was similar across patients in the 45–59, 60–89, and ≥90 ml/min eGFR subgroups. This was perhaps surprising, as it was expected that a greater incidence of cardiovascular comorbidities such as structural heart disease, hypertension, valvular heart disease, and diabetes in the more renally impaired subgroups would result in greater cumulative incidence of AF/AFL recurrence. However, it is important to recognize that the small populations in each subgroup, particularly in the most renally impaired group, may impact the generalizability of the results to a wider patient population. In addition, previous use of AADs, in particular amiodarone, was more common in patients with impaired renal function, indicating that these patients could also have more progressed forms of AF/AFL. These findings are reflective of the results obtained from the parent EURIDIS‐ADONIS trials in which dronedarone was shown to increase the median time to first AF/AFL recurrence versus placebo. Therefore, this analysis suggests that dronedarone is equally effective in renally impaired patients for treatment of AF/AFL as those without renal impairment.

There was a marked lowering of heart rate by 12–17 bpm during AF/AFL recurrences associated with dronedarone treatment compared with placebo regardless of the severity of renal impairment. This is consistent with a previous study that reported a heart rate reduction of 11.7 bpm with dronedarone versus placebo (*p* < .0001)^23^ and with the overall data from the EURIDIS‐ADONIS trials.^13^

Interestingly, there appeared to be an increase in left atrial anteroposterior diameter between the least and the most renally affected subgroups. This trend towards higher left atrial diameter with worse renal function has been reported previously and is associated with more rapid renal decline, increased likelihood of treatment resistance, greater contributory comorbidities, and increased all‐cause mortality.^24^, ^25^, ^26^, ^27^, ^28^ The data presented in this analysis demonstrate that the most renally impaired subgroup are also the oldest and present the largest mean left atrial anteroposterior diameter compared with younger patients with smaller left atrial size who demonstrate a higher likelihood of maintaining sinus rhythm during follow‐up.^26^ Whilst this may provide an explanation outside of statistical power as to why the most renally impaired subgroup did not demonstrate significantly increased time to first AF/AFL in the dronedarone versus placebo groups, AF/AFL events in the placebo groups of the least and most renally impaired subgroups were rather similar (80% in the 30–44 ml/min subgroup vs. 74% in the ≥90 ml/min subgroup), suggesting that the population age and atrial diameter were not significant factors in AF/AFL occurrence.

Competition of dronedarone with creatinine for cation transport channel secretion by renal tubules has been shown to result in a reversible significant reduction in creatinine clearance of about 18%.^29^ Whilst typically an indicator of reduced renal function, such inhibition of creatinine secretion has been demonstrated across a range of drugs,^30^ which is not necessarily associated with impaired renal function. Similarly, this analysis showed a numerical increase in serum creatinine in patients who received dronedarone, which was maintained until the end of the study. Whilst this is consistent with inhibition of creatinine secretion by dronedarone, which by creatinine‐derived glomerular filtration estimations would indicate a reduction in eGFR, it should be noted that in earlier studies no numerical effect of dronedarone on non‐creatinine derived GFR estimations was observed.^29^ Additionally, it is important to stress that the use of formulas to calculate eGFR based on creatinine level may underestimate renal function following treatment with dronedarone due to various confounders (e.g., muscle mass and dehydration).^31^, ^32^ If problematic, creatinine‐derived eGFR results can be combined with clinical assessment of cystatin C levels for the most accurate results, although it should be noted that this is a more costly approach.^31^, ^32^

No threshold was identified at which dronedarone efficacy or its safety profile was negatively impacted. Neither were any effects observed that might suggest alternative drug interactions introduced by the renal activity of dronedarone. No significant differences in adverse events were observed between any eGFR subgroups, suggesting that renal function does not impact the safety profile of dronedarone.^13^

The analysis has some limitations. Primarily, the stratification of patients from the EURIDIS‐ADONIS trials into subgroups according to renal function was not predefined and hence resulted in a loss of statistical power, particularly, in the most renally impaired subgroup. Additionally, having smaller patient groups may have increased the impact of confounders when comparing between eGFR strata, potentially influencing outcomes. As with many trials investigating AF/AFL at that time, continuous monitoring was not performed. It is, therefore, possible that some recurrent arrhythmia events may have been missed, particularly as the majority of reported events were symptomatic AF/AFL, despite AF/AFL recurrences typically being asymptomatic.^13^ Creatinine levels were not systematically collected after the end of the EURIDIS‐ADONIS trials, so data on the reversibility of elevated creatinine values are not available in this particular study population.

In conclusion, this post hoc analysis helps address the paucity of information regarding the use of AADs to treat patients with both AF/AFL and CKD. Stratification of patients into subgroups based on renal impairment demonstrated that dronedarone may provide an effective therapeutic option with an acceptable safety profile for people with AF/AFL and impaired renal function. Considering the data presented by this analysis, dronedarone can be considered a viable treatment option for eligible patients with renal function ≥45 ml/min, without the need for dose adjustment or continuous monitoring of renal function. However, further studies with larger patient populations, stratifying patients according to their renal function status, would be required to confirm the efficacy and safety of dronedarone in patients with impaired renal function.

## CONFLICT OF INTERESTS

Wojciech Zareba has received research grants from Gilead Sciences, LivaNova, Biotronic, and has consulted for AstraZeneca, MyoKardia, Abbott, and Medtronic. Dan Atar has received honoraria from Boehringer‐Ingelheim, Bayer, BMS/Pfizer, AstraZeneca, MSD, Sanofi, Amgen, and Novartis. Jun Zhu has acted as a speaker for Sanofi. J Reiffel has served as an investigator for Medtronic, Janssen, and Sanofi; and as a consultant for Medtronic, Sanofi, Acension, Correvio, and Amarin during the past 12 months. Peter Kowey has served as a consultant for Sanofi. Ulf Ludwigs, Mattias Wieloch, and John Stewart are employees of Sanofi and may hold shares and/or stock options in the company. The remaining authors declare that there are no conflict of interests.

## AUTHOR CONTRIBUTIONS

*Conceptualization*: Munveer Thind, Peter Kowey, Ulf Ludwigs, and Mattias Wieloch. *Data curation*: John Stewart. *Formal analysis*: John Stewart. *Investigation*: Munveer Thind, Wojciech Zareba, Dan Atar, Harry J. G. M. Crijns, Jun Zhu, Hui‐Nam Pak, James Reiffel, and Peter Kowey. *Methodology*: Munveer Thind, Wojciech Zareba, Dan Atar, Harry J. G. M. Crijns, Jun Zhu, Hui‐Nam Pak, James Reiffel, Ulf Ludwigs, Mattias Wieloch, John Stewart, and Peter Kowey. *Validation*: John Stewart. *Visualization*: John Stewart. *Writing—original draft*: Fishawack Health. *Writing—review & editing*: Munveer Thind, Wojciech Zareba, Dan Atar, Harry J. G. M. Crijns, Jun Zhu, Hui‐Nam Pak, James Reiffel, Ulf Ludwigs, Mattias Wieloch, John Stewart, and Peter Kowey.

## Supporting information

Supporting information.

## Data Availability

Qualified researchers may request access to patient‐level data and related study documents including the clinical study report, study protocol with any amendments, blank case report form, statistical analysis plan, and data set specifications. Patient‐level data will be anonymized and study documents will be redacted to protect the privacy of our trial participants. Further details on Sanofi's data‐sharing criteria, eligible studies, and process for requesting access can be found at https://www.clinicalstudydatarequest.com/.
