# Supplementary material for: Efficacy and safety of dronedarone versus placebo in patients with atrial fibrillation stratified according to renal function: Post hoc analyses of the EURIDIS‐ADONIS trials
Source: Clin Cardiol. 2022 Jan 12;45(1):101–9. doi: 10.1002/clc.23765 (PMC8799050; doi:10.1002/clc.23765)
Supplement: Supplementary file 1 — Supporting information. [file CLC-45-101-s001.docx]

# SUPPLEMENTARY MATERIALS

EURIDIS-ADONIS eligibility criteria

Criteria for patient selection included male or female patients ≥21 years of age, who have had a minimum of 1 episode of AF/AFL in the preceding 3 months (documented by electrocardiography), and to be in sinus rhythm for at least 1 hour before randomization. Exclusion criteria encompassed patients demonstrating permanent AF/AFL (duration of at least 12 months); women of childbearing age not using birth control; patients who have had torsades de pointes; patients with persistent bradycardia of less than 50 bpm; second-degree (or higher) atrioventricular block; a PR interval of 0.28 seconds or more; clinically significant sinus-node disease without an implanted pacemaker; patients taking class I or III antiarrhythmic agents, patients with New York Heart Association class III or IV congestive heart failure; a serum creatinine level of 1.7 mg/dL (150 μmol/L) or more; severe electrolyte abnormalities; and clinically significant hepatic, pulmonary, endocrine, or other disorders associated with atrial fibrillation. Written informed consent was obtained from all patients.

Supplementary Table 1A. Summary of the unadjusted analysis of time from randomization to first AF/AFL recurrence within 12 months by creatinine clearance categories using MDRD formula - randomized and treated patients population

| Study | Treatment | Statistics | 30–44 mL/min  (n=49) | 45–59 mL/min  (n=320) | 60–89 mL/min  (n=766) | ≥90 mL/min  (n=96) |
| --- | --- | --- | --- | --- | --- | --- |
|  | | | | | | |
| Pooled | Placebo (n=407) | Number of patients with adjudicated first AF/AFL recurrence within 12 months from randomization | 12 | 74 | 186 | 29 |
|  | | | | | | |
|  |  | Median time in days (95% CI) | 42 (3;300) | 63 (20;109) | 53 (16;90) | 26 (7;183) |
|  | | | | | | |
|  | Dronedarone 800 mg (n=824) | Number of patients with adjudicated first AF/AFL recurrence within 12 months from randomization | 18 | 135 | 336 | 27 |
|  | | | | | | |
|  |  | Median time in days (95% CI) | 188 (7;NE) | 138 (90;244) | 90 (56;122) | 329 (120;NE) |
|  | | | | | | |
|  |  | Relative risk (Dronedarone/Placebo)^1^ | 0.685 | 0.680 | 0.801 | 0.594 |
|  | | | | | | |
|  |  | 95% CI^1^ | 0.330 ; 1.422 | 0.512 ; 0.903 | 0.670 ; 0.959 | 0.350 ; 1.007 |
|  | | | | | | |
|  |  | Log-rank's test result (p-value) | 0.2976 | 0.0067 | 0.0137 | 0.0454 |
| ^1^ Determined from Cox regression model. AF/AFL, atrial fibrillation/flutter; CI, confidence interval; MDRD, modification of diet in renal disease; NE, not enough data. | | | | | | |

Supplementary Table 1B. Summary of the unadjusted analysis of time from randomization to first AF/AFL recurrence within 12 months by creatinine clearance categories using CKD-EPI formula - randomized and treated patients population

| Study | Treatment | Statistics | 30 - 44 mL/min  (N=70) | 45 - 59 mL/min  (N=333) | 60 - 89 mL/min  (N=722) | >=90 mL/min  (N=104) |
| --- | --- | --- | --- | --- | --- | --- |
|  | | | | | | |
| Pooled | Placebo (N=406) | Number of patients with adjudicated first AF/AFL recurrence within 12 months from randomization | 16 | 72 | 181 | 32 |
|  | | | | | | |
|  |  | Median time in days (95% CI) | 59 (5;150) | 77 (23;122) | 35 (15;85) | 19 (5;102) |
|  | | | | | | |
|  | Dronedarone 800 mg (N=823) | Number of patients with adjudicated first AF/AFL recurrence within 12 months from randomization | 29 | 144 | 313 | 29 |
|  | | | | | | |
|  |  | Median time in days (95% CI) | 190 (20;NE) | 125 (87;210) | 84 (51;121) | NE (150;NE) |
|  | | | | | | |
|  |  | Relative risk (Dronedarone/Placebo)^1^ | 0.620 | 0.752 | 0.804 | 0.473 |
|  | | | | | | |
|  |  | 95% CI^1^ | 0.336 ; 1.144 | 0.567 ; 0.999 | 0.669 ; 0.965 | 0.285 ; 0.787 |
|  | | | | | | |
|  |  | Log-rank's test result (p-value) | 0.1177 | 0.0456 | 0.0174 | 0.0026 |
| ^1^ Determined from Cox regression model | | | | | | |


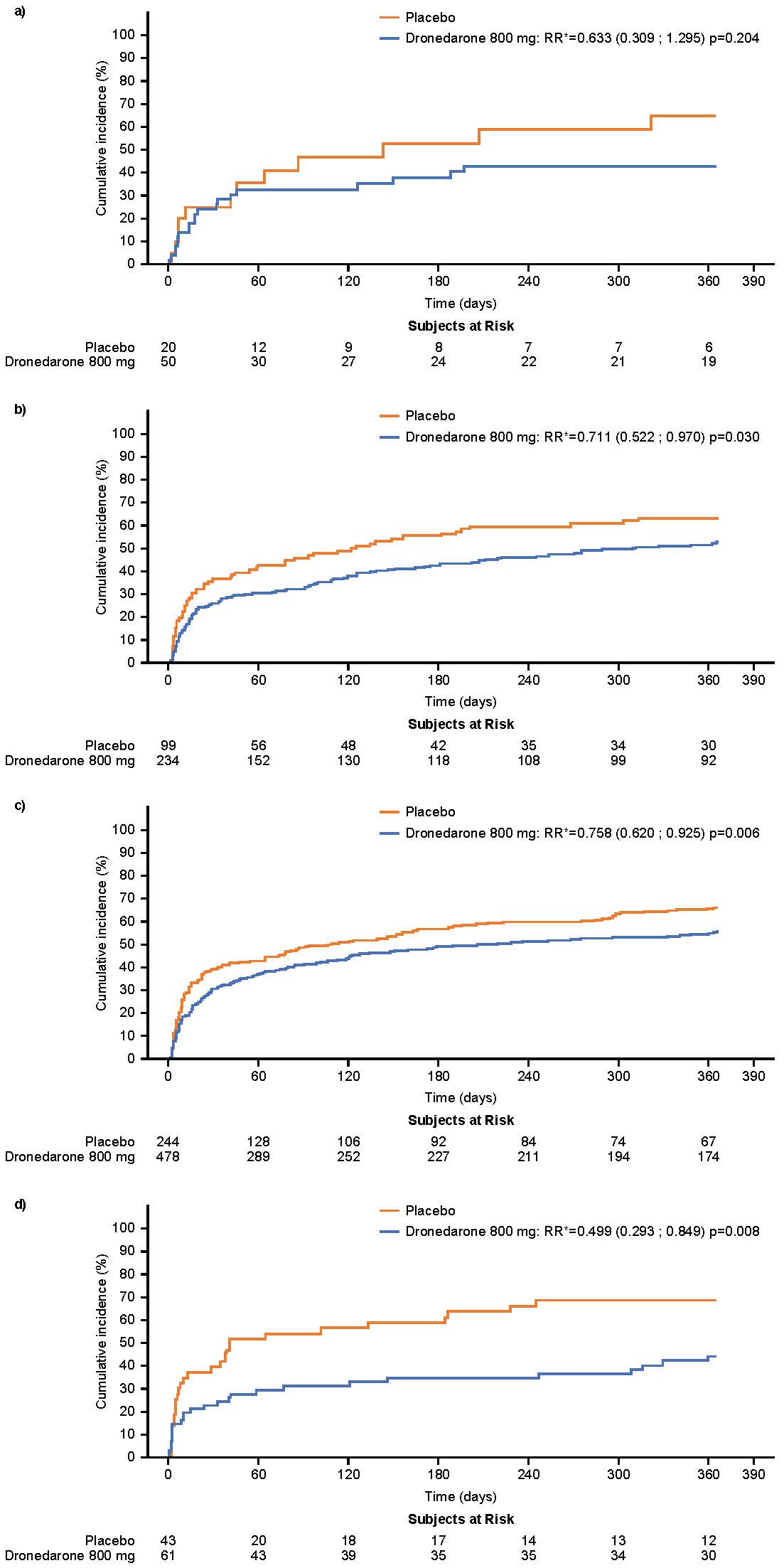
Supplementary Figure 1. Kaplan–Meier cumulative incidence of first symptomatic atrial fibrillation/flutter recurrence by eGFR category A) 30-44 mL/min, B) 45-59 mL/min, C) 60-89 mL/min and D) ≥90 mL/min

*RR values determined by Cox regression model. eGFR, estimated glomerular filtration rate
